# Quantification of lactoyl-CoA (lactyl-CoA) by liquid chromatography mass spectrometry in mammalian cells and tissues

**DOI:** 10.1098/rsob.200187

**Published:** 2020-09-23

**Authors:** Erika L. Varner, Sophie Trefely, David Bartee, Eliana von Krusenstiern, Luke Izzo, Carmen Bekeova, Roddy S. O'Connor, Erin L. Seifert, Kathryn E. Wellen, Jordan L. Meier, Nathaniel W. Snyder

**Affiliations:** 1 Center for Metabolic Disease Research, Department of Microbiology and Immunology, Temple University Lewis Katz School of Medicine, Philadelphia, PA 19140, USA; 2 Department of Cancer Biology and Abramson Family Cancer Research Institute, University of Pennsylvania Perelman School of Medicine, Philadelphia, PA 19104, USA; 3 Chemical Biology Laboratory, National Cancer Institute, Frederick, MD 21702, USA; 4 MitoCare Center, Department of Pathology, Anatomy, and Cell Biology, Thomas Jefferson University, Philadelphia, PA 19107, USA; 5 Department of Pathology and Laboratory Medicine, Perelman School of Medicine at the University of Pennsylvania, Philadelphia, PA 19104, USA; 6 Center for Cellular Immunotherapies, Perelman School of Medicine at the University of Pennsylvania, Philadelphia, PA 19104, USA

**Keywords:** metabolism, lactoyl-CoA, lactyl-CoA, high resolution, LC-HRMS

## Abstract

Lysine lactoylation is a recently described protein post-translational modification (PTM). However, the biochemical pathways responsible for this acylation remain unclear. Two metabolite-dependent mechanisms have been proposed: enzymatic histone lysine lactoylation derived from lactoyl-coenzyme A (lactoyl-CoA, also termed lactyl-CoA), and non-enzymatic lysine lactoylation resulting from acyl-transfer via lactoyl-glutathione. While the former has precedent in the form of enzyme-catalysed lysine acylation, the lactoyl-CoA metabolite has not been previously quantified in mammalian systems. Here, we use liquid chromatography–high-resolution mass spectrometry (LC-HRMS) together with a synthetic standard to detect and validate the presence of lactoyl-CoA in cell and tissue samples. Conducting a retrospective analysis of data from previously analysed samples revealed the presence of lactoyl-CoA in diverse cell and tissue contexts. In addition, we describe a biosynthetic route to generate ^13^C_3_^15^N_1_-isotopically labelled lactoyl-CoA, providing a co-eluting internal standard for analysis of this metabolite. We estimate lactoyl-CoA concentrations of 1.14 × 10^−8^ pmol per cell in cell culture and 0.0172 pmol mg^−1^ tissue wet weight in mouse heart. These levels are similar to crotonyl-CoA, but between 20 and 350 times lower than predominant acyl-CoAs such as acetyl-, propionyl- and succinyl-CoA. Overall our studies provide the first quantitative measurements of lactoyl-CoA in metazoans, and provide a methodological foundation for the interrogation of this novel metabolite in biology and disease.

## 1. Introduction

Acyl-coenzyme A (acyl-CoA) thioesters serve as critical metabolites in cellular energy metabolism. They also play a role in cellular signalling, acting as the acyl-donors for enzymatic and non-enzymatic PTMs collectively known as lysine acylation. Several studies have found that lysine acylation is sensitive to cellular metabolism, implying that acyl-CoA levels may directly link metabolism to PTM-mediated signalling [1]. This has the potential to occur either via the utilization of acyl-CoAs as rate-limiting cofactors in enzymatic protein modifications catalysed by lysine acyl-transferase (KAT) and histone acyltransfease (HAT) enzymes, or through non-enzymatic acylation reactions that can be heavily influenced by the electrophilicity of individual acyl-CoA species [2]. A physiochemically diverse set of acylations have been detected on histone lysine residues, including not only the predominant acetylation but also succinylation, propionylation, (iso)butyrylation, crotonylation, malonylation and several others [3]. Lysine acylations generally correlate closely with the abundance of corresponding acyl-CoA [4].

Recently, Zhang *et al.* [5] reported a new addition to this emerging class of PTMs, known as lysine lactoylation. Applying antibody-based enrichment, this PTM was mapped to histone in M1 polarized macrophages and shown to correlate with altered gene expression during immune activation. Furthermore, lysine lactoylation within *in vitro* transcription assays was found to be dependent on both the presence of a KAT enzyme (p300) as well as lactoyl-CoA, consistent with enzymatic acyl-transfer of the lactate group from the acyl-CoA to histones. Since lactate concentrations can shift from less than 1 mM to greater than 20 mM across both normal and pathological processes [6], a biochemical link between lactate and histone modifications would be of interest in fields ranging from exercise physiology to cancer metabolism.

While microbial lactoyl-CoA production has been engineered in bio-industrial applications [7], the existence or quantification of lactoyl-CoA (also called lactyl-CoA and 2-hydroxypropanoyl-CoA) in mammalian cells or tissues has not been previously reported in the literature. Furthermore, the recent discovery of a parallel route to lysine lactoylaton via non-enzymatic acyl-transfer from lactoyl-glutathione raises the question as to whether these two pathways play distinct or similar roles [8]. Since there is no described lactoyl-CoA synthetase or transferase that activates lactate to lactoyl-CoA in mammalian biochemistry, the *in vitro* and *in vivo* role of lactoyl-CoA remains poorly described. In order to better understand the potential role of enzyme-catalysed lysine lactoylation in metazoans, here we report a method for the detection of lactoyl-CoA by liquid chromatography–high-resolution mass spectrometry (LC-HRMS). Fortuitously, we find that lactoyl-CoA derivatives containing ^13^C_3_^15^N_1_-pantothenate are formed in human cells during stable isotope labelling by essential nutrients in cell culture (SILEC), providing access to an isotopically labelled internal standard for quantitative studies [9]. Applying this approach enables a comparative analysis of lactoyl-CoA and other metabolic acyl-CoAs, providing some initial insights into the concentration of this metabolite. By enabling the detection and quantitative analysis of lactoyl-CoA, our study provides the fundamental underpinnings for understanding the function of this metabolite in biology and disease.

## 2. Methods

### 2.1. Chemicals and reagents

5-sulfosalicylic acid (SSA), trichloroacetic acid and ammonium acetate were purchased from Sigma-Aldrich (St Louis, MO). Optima LC-MS grade methanol (MeOH), acetonitrile (ACN), formic acid and water were purchased from Fisher Scientific (Pittsburgh, PA). ^13^C_6_^15^N_2_-pantothenate calcium salt was purchased from Isosciences (Ambler, PA).

### 2.2. Synthesis of l-lactoyl-coenzyme

Synthesis was conducted as previously described with minor modifications [7]. Briefly, l-lactic acid (90 mg, 1.0 mmol) and *N*-hydroxy succimide (NHS) were dissolved in anhydrous tetrahydrofuran (THF, 8 ml). A solution of dicyclohexylcarbodiimide (206 mg, 1.0 mmol) in THF (20 μl) was added dropwise to the l-lactic acid solution at 22°C with stirring. The reaction mixture was stirred overnight at 22°C. The resulting precipitate was removed by vacuum filtration and the filtrate was condensed under reduced pressure to yield l-lactyl-NHS which was used without further purification.

A solution of l-lactyl-NHS (9.3 mg, 50 mmol) in methanol (0.5 ml) was added to a solution of coenzyme A (25 mg, 33 mmol) in aqueous bicarbonate solution (0.5 ml, 500 mM, pH 8) and stirred at 22°C for 3 h. The remaining aqueous solution was neutralized with trifluoroacetic acid (TFA, 20 ml) and immediately purified by C_18_-reversed-phase-silica chromatography. Method: (solvents—A: 0.05% TFA in water, B: acetonitrile) gradient—0–2 column volumes (CV), 0% B; 2–12 CV, 0–50% B; 12–13 CV, 50–100% B; and 13–25 CV, 100% B. Fractions containing the desired product were pooled and lyophilized to provide a fluffy white powder (7.3 mg, 26% yield).

### 2.3. Biosynthetic production of ^13^C_3_^15^N_1_-acyl-coenzyme libraries

To produce stable isotope labelled acyl-CoAs as internal standards, we used stable isotope labelling by essential nutrients in cell culture (SILEC) in either mammalian cell culture systems [9] or S. cerevisiae [10]. In both cases, ^13^C_3_^15^N_1_-pantothenate (as ^13^C_6_^15^N_2_-pantothenate calcium salt) replaced unlabelled pantothenate in cell culture media over sufficient generations to incorporate into the pantothenate-derived moiety within CoA to achieve greater than 99.5% labelling. For some less abundant acyl-CoAs, the addition of the free carboxylic acid was necessary to produce the labelled acyl-CoA [11]. The labelled cells were harvested in 10% (w/v) trichloroacetic acid for short-chain acyl-CoA or flash frozen as a cell pellet for full acyl-chain length extraction and the procedure for this biosynthetic production and use is detailed elsewhere in a full protocol [12].

### 2.4. Liquid chromatography-high-resolution mass spectrometry

Acyl-CoAs were quantified by LC-HRMS as previously published [10,13]. For quantification from cells, media was aspirated from attached cells, 1 ml of ice-cold 10% (w/v) trichloroacetic acid (Sigma-Aldrich) in water was added, and cells were scraped and transferred to 1.5 ml Eppendorf tubes. Samples were spiked with 50 µl internal standard prepared as previously published [10] sonicated for 12 × 0.5 s pulses in, then protein was pelleted by centrifugation at 17 000*g* from 10 min at 4°C. The cleared supernatant was purified by solid-phase extraction using Oasis HLB 1cc (30 mg) SPE columns (Waters). Columns were washed with 1 ml methanol, equilibrated with 1 ml water, loaded with the sample, desalted with 1 ml water and eluted with 1 ml methanol containing 25 mM ammonium acetate. Samples were also extracted by addition of 1 ml −80°C 80 : 20 methanol : water, followed by sonication and centrifugation as above then evaporation of the supernatant. A retrospective analysis was also conducted on tissue samples using the extraction method of Minkler *et al*. [14] modified for isotope dilution LC-MS [10]. Calibration curves were prepared using analytical standards from Sigma-Aldrich (for all acyl-CoAs other than lactoyl-CoA) and processed identically to the samples.

The purified extracts were evaporated to dryness under nitrogen then suspended in 55 µl 5% (w/v) 5-sulfosalicylic acid in optima HPLC grade water. Five microlitres of samples in 5% SSA were analysed by injection of an Ultimate 3000 HPLC coupled to a Q Exactive Plus (Thermo Scientific) mass spectrometer in positive ESI mode using the settings described previously [13], or for the full acyl-length extraction [11]. Modifications were made to these methods for MS/HRMS experiments as described in each experiment for the acquisition of precursor and product ions. Data were integrated using XCalibur Quan and Qual Browsers (Thermo Scientific), Tracefinder v. 4.1 (Thermo Scientific) software, and additional statistical analysis conducted by Prism v. 7.05 (GraphPad).

### 2.5. Cell culture

Cells were passaged every 2–3 days at 80% confluence. Human hepatocellular carcinoma (HepG2) cells were used at less than 20 passages from ATCC stocks (ATCC) and were cultured in high-glucose DMEM (Thermo-Fisher Scientific), supplemented with 10% fetal bovine serum (Gemini Biosciences) and penicillin/streptomycin (Thermo-Fisher Scientific). All cells were tested mycoplasma-free.

### 2.6. Heart and muscle tissue analysis

Experiments were performed on 11- to 13-week-old male and female C57BL/6 J mice. Mice were maintained on a 12–12 h light–dark cycle (lights on: 7:00 to 19:00) and ad libitum fed a standard diet (LabDiet 5001, Purina). Fasted mice were fasted overnight (19:00 to 9:00). Whole heart samples were always harvested in the morning, snap frozen and stored at −80°C.

## 3. Results

### 3.1. Synthesis of lactoyl-coenzyme

**Figure 1.**
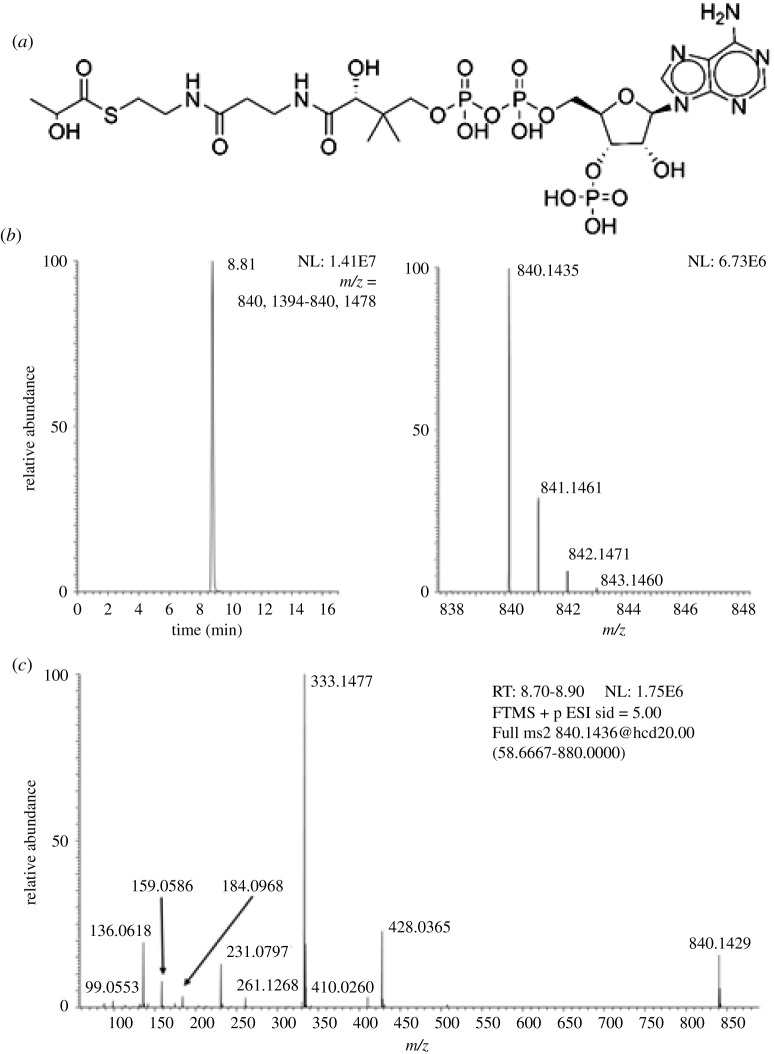
(*a*) Structure of lactoyl-CoA (stereochemistry not shown). (*b*) LC-HRMS of synthetic lactoyl-CoA. (*c*) LC-MS/HRMS of lactoyl-CoA.

To define detection and identification of lactoyl-CoA we synthesized a pure standard. The resulting product was a white crystalline solid. Both NMR and LC-HRMS of the pure standard provided evidence of the structure of lactoyl-CoA (figure 1). ^1^H NMR (500 MHz, D_2_O) *δ* 8.56 (d, *J* = 5.7 Hz, 1H), 8.35 (s, 1H), 6.13 (d, *J* = 5.2 Hz, 1H), 4.81 (s, 2H), 4.52 (s, 1H), 4.29 (q, *J* = 7.0 Hz, 1H), 4.19 (s, 2H), 3.94 (s, 1H), 3.79 (d, *J* = 9.3 Hz, 1H), 3.54 (d, *J* = 9.2 Hz, 1H), 3.37 (t, *J* = 6.7 Hz, 2H), 3.26 (t, *J* = 6.5 Hz, 2H), 2.35 (t, *J* = 6.7 Hz, 2H), 1.26 (d, *J* = 7.0 Hz, 3H), 0.86 (s, 3H), 0.74 (s, 3H). For HRMS, we observed an intense [MH]+ ion (*m/z* = 840.1435) matching the theoretical *m/z* calculated for C_24_H_41_N_7_O_18_P_3_S+ (840.1436138) (Δppm = −0.135). Based on previous literature of the stability of acyl-CoAs in acid and lowered temperature avoiding excessive freeze–thaw cycles [10], we re-suspended the product in 10% TCA in water at 1 mg ml^−1^ to generate aliquots of concentrated stocks for future use.

### 3.2. Qualitative characterization of lactoyl-CoA in cells and tissues

**Figure 2.**
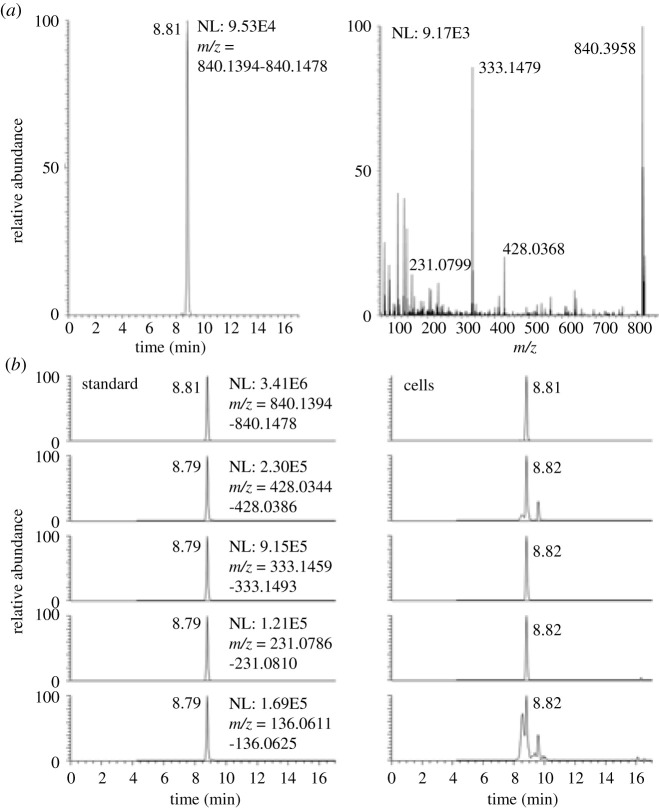
(*a*) LC-HRMS of lactoyl-CoA from HepG2 cell extract and (*b*) LC-MS/HRMS of synthetic lactoyl-CoA (left) and the same ions in cell extract (right).

Using LC-HRMS and LC-MS/HRMS, we detected lactoyl-CoA from cells. The cell derived lactoyl-CoA matched the synthetic standard on retention time, HRMS and MS/HRMS on major product ions (figure 2). By contrast, the extraction of acyl-CoAs from a different 10 cm plate of cells with protein precipitation by −80°C 80:20 methanol : water did not yield a discernable peak above noise (data not shown). The retention time of the putative lactoyl-CoA (tr = 8.8 min) in reversed-phase chromatography fell earlier than propionyl-CoA (tr = 9.2) corresponding to the additional hydroxyl group in the acyl-group.

Since acyl-CoAs produce a wide variety of product ions upon collision-induced dissociation, we examined the likely specificity of each major product ion via plotting chromatograms for the top four ions derived from lactoyl-CoA (figure 2). Within cells, the product ion corresponding to the M-507 (M-506.99575) neutral loss (*m/z* 333.14786) was the most intense and specific.

**Figure 3.**
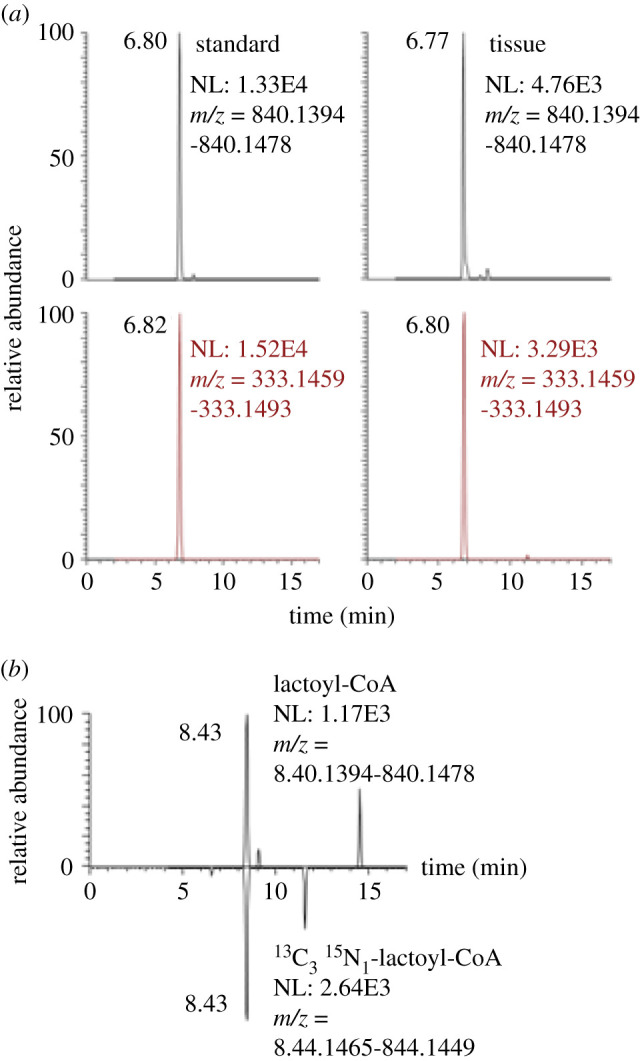
Detection of lactoyl-CoA from retrospective analysis (*a*) of tissues by LC-HRMS (top) and LC-MS/HRMS (bottom) and (*b*) co-elution of lactoyl-CoA with ^13^C_3_^15^N_1_-lactoyl-CoA from retrospective data.

Based on the reanalysis of data from samples previously acquired by LC-HRMS, we examined the detection of lactoyl-CoA in other cellular and tissue contexts. Since our routine methods of acyl-CoA analysis include a full scan acquisition covering the [MH]+ ion, we predominantly detected from the synthetic standard, this allowed semi-quantitative retrospective analysis of previously acquired data. Additional experiments using a mixed chain length acyl-CoA extraction from Minkler *et al*. [14] similarly detected a LC-HRMS and LC-MS/HRMS peak corresponding to lactoyl-CoA from heart and muscle samples previously acquired, which we then matched to a standard acquired with the same chromatographic method (figure 3).

From previous stable isotope labelling using essential nutrients in cell culture where acyl-CoA libraries incorporating ^13^C_3_^15^N_1_-pantothenate at high efficiency (greater than 99.5%) were generated and spiked into samples, we interrogated generation and co-elution of ^13^C_3_^15^N_1_-labelled lactoyl-CoA with unlabelled lactoyl-CoA (figure 3). Previous studies using pantothenate SILEC in HepG2 cells demonstrated co-elution of lactoyl-Coa with ^13^C_3_^15^N_1_-labelled lactoyl-CoA. However, we found unfortunately that the production of acyl-CoA internal standards by yeast SILEC in the conditions we commonly used did not produce detectable amounts of ^13^C_3_^15^N_1_-labelled lactoyl-CoA. This lack of ^13^C_3_^15^N_1_-lactoyl-CoA in these samples strongly suggests that there is no post-extraction acyl-transfer of lactate to CoASH, as the SILEC standard includes ^13^C_3_^15^N_1_-CoASH that would have artificially formed ^13^C_3_^15^N_1_-lactoyl-CoA if possible.

### 3.2. Partial re-validation of a quantitative method to include lactoyl-coenzyme

Extending previously published quantitative methodology for acyl-CoA quantification for short- and longer chain acyl-CoAs [13], we partially revalidated the LC-HRMS method for select quantitative properties of lactoyl-CoA. The linear range was estimated for isotope dilution and label-free quantification. In both cases, lactoyl-CoA was linear (*R*^2^ > 0.998) and observed values within 25% of expected values from 0.03 pmol per sample (3 fmol on column) to the highest range tested at 500 pmol per sample (50 fmol on column). Lactoyl-CoA was detectable down to 0.002 pmol per sample (0.2 fmol on column), but quantification was unstable across runs over 24 h. Re-injection of samples from both purified standards, and purified standards spiked into cellular extract were stable (±5%) over 24 h in a chilled autosampler. Unfortunately, as noted, our method of producing internal standards in yeast did not generate a lactoyl-CoA under the conditions we used. Thus, we investigated using surrogate internal standards including ^13^C_3_^15^N_1_-acetyl-, ^13^C_3_^15^N_1_-succinyl- and ^13^C_3_^15^N_1_-HMG-CoA. All produced linear calibration curves with high R^2^ values (greater than 0.998) when plotting the ratios of analyte/each internal standard as the dependent variable and similar deviation of calibration points from observed versus expected values.

### 3.3. Lactoyl-coenzyme is in low abundance relative to other commonly quantified short-chain acyl-CoAs

We quantified lactoyl-CoA via relative response factor to other short-chain acyl-CoAs for retrospective sample analysis. This allowed us to rank the molar amount of lactoyl-CoA versus other acyl-CoAs detected in cells. We calculated proportionality constants using known amounts of lactoyl-CoA across a calibration curve of serial dilutions of cell and tissue relevant concentrations of other short-chain acyl-CoAs. Proportionality constants on a molar basis were calculated as

$$k=\frac{\left[\text{AUC}(\text{lactoyl}\text{-}\text{CoA})/\text{pmol}(\text{lactoyl}\text{-}\text{CoA})\right]}{\left[\text{AUC}(\text{acyl}\text{-}\text{CoA})/\text{pmol}(\text{acyl}\text{-}\text{CoA})\right]}.$$

Propionyl-CoA and succinyl-CoA had the response factors that most closely approximated 1, where the same molar amount of lactoyl-CoA and other acyl-CoAs generated the same intensity of signal. Thus, we used propionyl-CoA as a surrogate to retrospectively estimate lactoyl-CoA concentrations in a semi-quantitative manner. We found that lacotyl-CoA in HepG2 cells was quantifiable at 0.011 pmol 10^−6^ cells, comparable to crontonyl-CoA (0.033 pmol 10^−6^ cells), but markedly less abundant (30–250 times lower) than major acyl-CoAs such as acetyl-, succinyl- and propionyl-CoA (table 1).

**Table 1.** Concentrations of short-chain acyl-CoAs within HepG2 cells.

acyl-CoA	pmol/10^6^ cells	standard deviation
acetyl-CoA	10.644	1.364
succinyl-CoA	25.467	2.818
propionyl-CoA	3.532	0.652
CoASH	1.734	0.189
butyryl-CoA	1.013	0.159
HMG-CoA	0.971	0.326
glutaryl-CoA	0.647	0.112
valeryl-CoA	1.118	0.143
crotonoyl-CoA	0.032	0.015
lactoyl-CoA	0.011	0.003

Since we could also detect lactoyl-CoA in tissues, we performed the same semi-quantitative estimation in heart tissue from mice. In these experiments, since we did not have ^13^C_3_^15^N_1_-lactoyl-CoA due to the method of production of the internal standard, we also used ^13^C_3_^15^N_1_-propionyl-CoA as a surrogate internal standard based on the similarity in response generated from equimolar amounts found above. We compared the lactoyl-CoA concentration of murine heart by sex and by fed/fasted status. Across 25 mouse samples of murine heart, we retrospectively estimated a mean (standard deviation) of 0.0179 (0.0153) pmol mg^−1^ tissue wet weight. We could detect no difference between fasted (0.0187 pmol mg^−1^) or fed (0.0172 pmol mg^−1^). Similarly, we found no difference between lactoyl-CoA content in hearts from male (0.0214 (0.0173) pmol mg^−1^) and female (0.0145 (0.0119) pmol mg^−1^) mice in this experiment. In these same tissues, acetyl-CoA was 5.77 (3.08) pmol mg^−1^ and propionyl-CoA 0.476 (0.224) pmol mg^−1^. Thus, again we found lactoyl-CoA to be 335 (versus acetyl-) and 27 (versus propionyl-) times lower than major short-chain acyl-CoAs, but still detectable.

## 4. Discussion

Increasingly sensitive proteomics approaches discover and describe a large abundance of protein PTMs. These PTMs are commonly putatively attributed to acyl-transfer from various metabolites including biologically activated or reactive metabolite classes including acyl-CoA thioesters [1]. Due to the shared physiochemical properties of these classes of metabolites, methods of purification, separation and analysis for certain members of these classes of metabolites tend to also capture other members of the same metabolite family. This becomes increasingly evident with methods of detection including HRMS that can use less restrictive analyte detection. This leads to a rise of a combination of targeted and untargeted metabolomics, termed hybrid metabolomics where a limited targeted set of analytes is profiled, but metabolomics level interrogation on the background data can still be performed [15]. As we demonstrate here, the re-interrogation of previously acquired background metabolomics data can be useful for metabolite discovery and semi-quantification. This was especially useful because our approach to internal standardization using the SILEC approach, generated a library of stable isotope labelled internal standards that fortuitously included ^13^C_3_^15^N_1_-lactoyl-CoA. Other approaches to internal standard production from isotope labelled biological sources may also show this same benefit [16]. However, the yeast-based method of SILEC production used here did not produce detectable ^13^C_3_^15^N_1_-lactoyl-CoA which is unfortunate for quantification purposes but does indicate that acyl-transfer post-extraction did not result in an artefactual lactoyl-CoA generation. Such an artefact is certainly not unprecedented, as acyl-groups are known to migrate to and from CoASH, and transacylation of carboxyclic acid-containing acyl groups is biologically and pharmacologically common [17].

Our data do suggest that lactoyl-CoA generation is relatively less abundant than other acyl-CoAs. This is unsurprising as histone lysine lactoylation was reported in conditions including hypoxia and M1 macrophage polarization where lactate production is high [5]. Thus, there is likely a precedent for contexts where lacotyl-CoA would be more abundant. The quantitative comparison to crontonyl-CoA, which is also thought to be the acyl-donor for histone crontoylation, may put these modifications in the same realm of biological plausibility [18]. However, our retrospective analysis did not detect high levels of lactoyl-CoA in conditions where we expected it. This included cell culture within incubation of exogenous lactate in T-cell culture (data not shown). The reasons for this discrepancy across detection require systematic investigation in the future. Similarly, investigations of the comprehensive changes of other acyl-CoAs (including CoASH) that could compete with lactoyl-CoA for the formation of acyl-transfer to lysine would be warranted.

This study has two important limitations that warrant discussion. First, although the description of lyisine lactoylation was the reason we synthesized then searched for lactoyl-CoA this study does not quantify either lactoyl-lysine or lacotyl-GSH. Future studies will be needed to compare the relative concentrations of each of these acylated molecules and the biological contexts where they may be important. As noted recently by Kulkarni and Brookes, Kla would be isobaric to *N^6^*-carboxyethyl lysine resulting from methyglyoxal, a reactive side-product of glucose metabolism [19]. Thus, future studies must resolve these potentially interfering species via isotope tracing or detection methods capable of discerning the two lysine modifications. Second, our methods were operating near their estimated limits of detection for lactoyl-CoA which poses problems of censoring and imposes limits on experimental design. Further improvements in analysis, including more sensitive LC-MS approaches reaching atto- and zepto-mole on column quantification may be needed considering the low abundance of lactoyl-CoA. Increased sensitivity would be especially necessary for isotopic tracing applications where additional sensitivity is required since labelling distributes signal intensity across isotopologues. Such isotope tracing experiments from labelled glucose, pyruvate and lactate would be one of the most straightforward ways of establishing the route and mechanism of lactoyl-lysine formation and are tractable even in human studies [20]. Additionally, future methods could achieve separation of enantiomeric acyl-CoAs, including lactoyl-CoA (which could form from d- or l-lactic acid). These analytical improvements would be useful in the next steps of elucidating the as yet unknown biological function, enzymology and occurrence of lactoyl-CoA.

The biological implication of acyl-donors is that they can serve as a nutrient responsive sensor, especially when the acylation is a modification of histones that results in a functional change on transcription (or other processes involving histones) [1]. Ongoing work has implicated higher abundance acyl-CoAs, especially acetyl-CoA, in nutrient response. Notably, even the less abundant crotonyl-CoA that was the closest comparator in this study has similarly been implicated in transcriptional changes resulting from crontonylation of histones [18] and cellular effects *in vivo* with microbiota-dependent metabolite changes [21]. In the case where the acylations are enzymatically generated, lower abundance acyl-donating substrates may be limiting rather than the acyl-transferase enzymes. For non-enzymatically generated acylations, the substrate concentration and reactivity may be the most important driver of the acylation [2]. This poses a particular difficulty in finding, and then quantifying, those low abundance substrates. Alternatively, it has been proposed that the channelled or highly compartmentalized nature of some reactions involving moving acyl groups makes their concentration measurement at a whole-cell level less relevant to the underlying biology [22–24]. Thus, future work should also examine the concentration dependence and potential for compartmentalization and channelling of reactions using lactoyl-CoA. The ability to quantify this metabolite will enable these studies.

## 5. Conclusion

We can detect and quantify lactoyl-CoA within cells and tissues at low pmol abundance. This should be valuable for future studies interrogating the role of lactoyl-CoA and its mechanisms of production and use within wider biologic contexts. The utility of LC-HRMS data for retrospective reanalysis to examine newly described metabolites also highlights the power of this approach over more targeted acquisition strategies.

## Data accessibility

This article has no additional data.
